# Data set on the characterization of the phytoestrogenic extract and isolated compounds of the roots of *Inula racemosa* Hook F (Asteraceae)

**DOI:** 10.1016/j.dib.2018.02.004

**Published:** 2018-02-08

**Authors:** Mangathayaru Kalachaveedu, Divya Raghavan, Srivani Telapolu, Sarah Kuruvilla, Balakrishna Kedike

**Affiliations:** a Faculty of Pharmacy, Sri Ramachandra University, Chennai, India; b SRU Center for Indian Systems of Medicine-Quality Assurance and Standardization, Central Research Facility, Sri Ramachandra University, Chennai, India; c Dept of Pathology, Madras Medical Mission, Chennai, India; d Captain Srinivasa Murty Drug Research Institute for Ayurveda and Siddha (CSMDRIAS) Ministry of Health & Family Welfare, Government of India, Chennai, India

**Keywords:** Alantolactone, Isoalantolactone, Stigmasterol glycoside, Inulin

## Abstract

The data presented in this article are related to the research article entitled ‘ Phyto estrogenic effect of *Inula racemosa* Hook. f – A cardio protective root drug in traditional medicine, (Mangathayaru K, Divya R, Srivani T et al., 2018) [1]. It describes the characterization details of the root extract and the compounds isolated from them that were shown to be phytoestrogenic *in vivo* and *in vitro* respectively.

**Specifications Table**

**Table:** 

Subject area	*Chemistry*
More specific subject area	*Chromatography, spectroscopy*
Type of data	*Image, Table*
How data was acquired	*HPTLC (CAMAG, Germany) NMR (Bruker AV-300 Supercon NMR system), IR ALPHA FT-IR (BrukerOptik, GmbH- Ettlingen, Germany)*
Data format	*Analyzed*
Experimental factors	*No pretreatment for characterization*
Experimental features	*HPTLC, IR, NMR*
Data source location	*Chennai, India*
Data accessibility	*Within the article*

**Value of the data**

• HPTLC standardization of *Inula racemosa* root extract based on inulin - a marker oligosaccharide, could be a blueprint for the characterization of polar extracts of Inula species.
• The Spectral data of Stigmasterol-3-O-β-D-glucopyranoside - reported first time from the root by the research paper, is data supportive of its characterization, a possible comparative data for its isolation from other species of Inula.
• IR and NMR spectra of alantolactone (ALT) and isoalantolactone (IALT) – investigated *invitro* for estrogenic activity, establish their identity.

## 1 Data

**Fig. 1:**
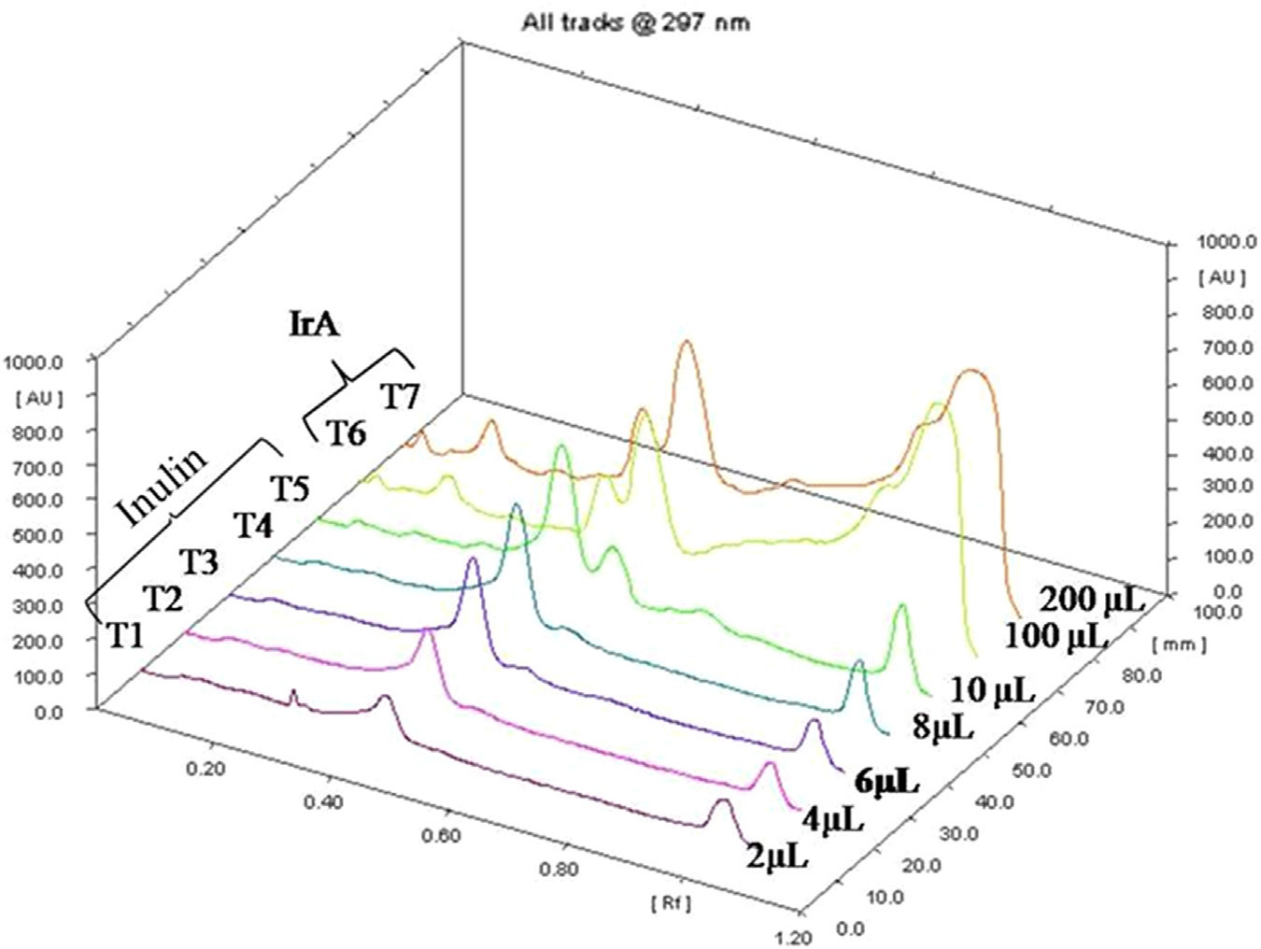
HPTLC densitometric quantification of inulin in methanolic extract of *Inula racemosa* (IrA) – HPTLC chromatogram.

This data describes the characterization of methanolic extract of *Inula racemosa* (IrA) roots using HPTLC (Fig. 1). The spectral assignments of compounds isolated from the root extract characterized using IR and NMR spectroscopy are also included (Table 1).

**Table 1:** Spectral assignments of Stigmasterol glucoside (SG), Alantolactone (ALT) and Isoalantolactone (IALT).

Compound	Spectral Assignment
SG	IR ν_max_ cm^−1^: 2917, 2855 (C-H stretching), 1736 (α,β- unsaturated γ- lactone), 1655 (-C=CH_2_), 1453 (-C=CH_2,_ δ C-H in plane), 1395,1340,1253 (C-O of lactone), 1160, 1123, 1038, 975, 920, 889 (-C=CH_2_, δ C-H out of plane), 852 (-C=CH, δ C-H, out of plane)
	^1^H NMR (δ, CDCl_3,_300 MHz) 1.04 (3H, d, J=7.0 Hz C-4 Me), 1.14 (3H, s, C-10 Me), 3.55 (1H, m, H-7), 4.77 (1H, m, H-8), 5.06( 1H, d, J=4.0 Hz, H-6), 5.60 and 6.13 (1H each, d, J=2.0 Hz, H-13)
	^13^C NMR (δ ppm) 41.6 (C-1), 22.5 (C-2), 32.5 (C-3), 37.4 (C-4), 148.8 (C-5), 118.7 (C-6), 39.4 (C-4), 148.8 (C-5), 118.7 (C-6), 39.4 (C-7), 76.3 (C-6), 42.5 (C-9), 32.6 (C-10), 170.3 (C-11), 139.7 (C-12), 121.5 (C-13), 16.6 (C-4, Me), 28.4 (C-10, Me).
ALT	IR ν_max_ cm^−1^: 2917, 2855 (C-H stretching), 1736 (α,β- unsaturated γ- lactone), 1655 (-C=CH_2_), 1453 (-C=CH_2,_ δ C-H in plane), 1395,1340,1253 (C-O of lactone), 1160, 1123, 1038, 975, 920, 889 (-C=CH_2_, δ C-H out of plane), 852 (-C=CH, δ C-H, out of plane)
	^1^H NMR (δ, CDCl_3,_300 MHz) 1.04 (3H, d, J=7.0 Hz C-4 Me), 1.14 (3H, s, C-10 Me), 3.55 (1H, m, H-7), 4.77 (1H, m, H-8), 5.06( 1H, d, J=4.0 Hz, H-6), 5.60 and 6.13 (1H each, d, J=2.0 Hz, H-13)
	^13^C NMR (δ ppm) 41.6 (C-1), 22.5 (C-2), 32.5 (C-3), 37.4 (C-4), 148.8 (C-5), 118.7 (C-6), 39.4 (C-4), 148.8 (C-5), 118.7 (C-6), 39.4 (C-7), 76.3 (C-6), 42.5 (C-9), 32.6 (C-10), 170.3 (C-11), 139.7 (C-12), 121.5 (C-13), 16.6 (C-4, Me), 28.4 (C-10, Me).
IALT	IR ν_max_ cm^−1^: 2929, 2836 (C-H stretching), 1761 (α, β- unsaturated γ- lactone), 1647 (-C=CH_2_), 1414 (-C=CH_2,_ δ C-H in plane), 1374, 1334, 1264 (C-O of lactone), 1139, 1103, 1036, 1013, 965, 891 (-C=CH_2_, δ C-H)
	^1^H NMR (δ, CDCl_3,_ 300 MHz) 0.81 (3H, s, C-10), 2.95 (1H, m, H-7), 4.41 and 4.74 (1H each, brs, C-4 – methylene), 4.48 (1H, m, H-8), 5.59 and 6.09 (1H each, brs, H-13)
	^13^C NMR (δ, CDCl_3,_ 75 MHz) 32.7 (C-1), 22.6 (C-2), 39.4 (C-3), 148.8 (C-4), 46.1 (C-5), 27.4 (C-6), 40.5 (C-7), 76.7 (C-8), 41.3 (C-9), 34.2 (C-10), 170.5 (C-11), 142.2 (C-12), 119.9 (C-13), 106.5 (C-4 methylene), 28.5 (C-10, Me).

## 2 Experimental design, materials and methods

### 2.1 Experimental materials

Inulin (92–95% purity) was purchased from Aumgene Biosciences Pvt Ltd (Gujarat, India). Precoated silica gel plates 60F_254_ of 0.2 mm thickness were from E Merck (Mumbai, India). Silica gel G 60–120 mesh for column chromatography was from SISCO Research (Mumbai, India).

### 2.2 Experimental design and methods

#### 2.2.1 HPTLC analysis of IrA

The extract of *Inula racemosa* was standardized for inulin using HPTLC analysis. The sample and inulin standard solutions were applied on pre-coated silica gel G 60 F254 (10 cm ×10 cm with 250 μm thickness, E. Merck) plate with a Hamilton 100 μl syringe using a Camag Linomat V applicator (automated spray-on applicator equipped with a 100 μl syringe and operated with the settings distance from the plate side edge 15 mm, and distance from the bottom of the plate 10 mm). The slit dimension was kept as 6.00 mm×0.45 mm. Linear ascending development was carried out in 10 cm×10 cm, Camag twin trough glass Chamber saturated with butanol: acetic acid: water (6.3:2.7:1) as mobile phase. After development, TLC plate was completely air dried at room temperature and derivatized with 20% sulphuric acid reagent. Peak areas for samples and standard were recorded by densitometric scanning at 297 nm, using a CAMAG TLC Scanner 3 with WINCATS version 3.2.1 software. Photodocumentation was performed using CAMAG REPROSTAR 3. The data of the peak areas were plotted against the corresponding concentrations. The obtained values were treated by linear regression analysis.

#### 2.2.2 Spectral characterization of isolated compounds

The IR spectra of the isolated compounds were taken on ALPHA FT-IR (BrukerOptik, GmbH- Ettlingen, Germany) Spectrometer equipped with a versatile high throughput ZnSe ATR crystal, using OPUS software version 6.5. Samples were scanned between 600 & 4000 cm^−1^.

^1^H and ^13^C NMR spectra were recorded on a Bruker AV-300 Supercon NMR system at 300 and 75 MHz respectively. Deuterated chloroform (CDCl_3_) was used as solvent with Trimethylsilane (TMS) as internal standard. Chemical shift values are given in δ scale with TMS as zero.
