# Supplementary material for: Data set on the characterization of the phytoestrogenic extract and isolated compounds of the roots of Inula racemosa Hook F (Asteraceae)
Source: Data Brief. 2018 Feb 8;17:770–3. doi: 10.1016/j.dib.2018.02.004 (PMC5988427; doi:10.1016/j.dib.2018.02.004)
Supplement: Supplementary file 1 — Supplementary material [file mmc1.pdf]

Conflicts of Interest Statement

Manuscript title: Data Supporting the characterization of Indrarasmasa Hookf root extract and its isolated compounds

The authors whose names are listed immediately below certify that they have NO affiliations with or involvement in any organization or entity with any financial interest (such as honoraria; educational grants; participation in speakers' bureaus; membership, employment, consultancies, stock ownership, or other equity interest; and expert testimony or patent-licensing arrangements), or non-financial interest (such as personal or professional relationships, affiliations, knowledge or beliefs) in the subject matter or materials discussed in this manuscript.

Author names: Kalachaveedu Mangathayaru
Divya Raghavan
Srivani Telapolu
Sarah Kuruvilla
Kedike Balakrishna

The authors whose names are listed immediately below report the following details of affiliation or involvement in an organization or entity with a financial or non-financial interest in the subject matter or materials discussed in this manuscript. Please specify the nature of the conflict on a separate sheet of paper if the space below is inadequate.

Author names:

None to disclose

Scanned by CamScanner

This statement is signed by all the authors to indicate agreement that the above information is true and correct (*a photocopy of this form may be used if there are more than 10 authors*):

Author's name (typed)	Author's signature	Date
Kalachaveedu Mangathayaru	[illegible]	21/12/17
Divya Raghavan	[illegible]	24/12/2017
Srivani Telapolu	Srivani	24/12/17
Sarah Kuruvilla	[illegible]	20-12-17
Kedike Balakrishna	K Balakrishna	21/12/17

Scanned by CamScanner
